# Supplementary figures and images for: Linking protein structural and functional change to mutation using amino acid networks
Source: PLoS One. 2022 Jan 21;17(1):e0261829. doi: 10.1371/journal.pone.0261829 (PMC8782487; doi:10.1371/journal.pone.0261829)

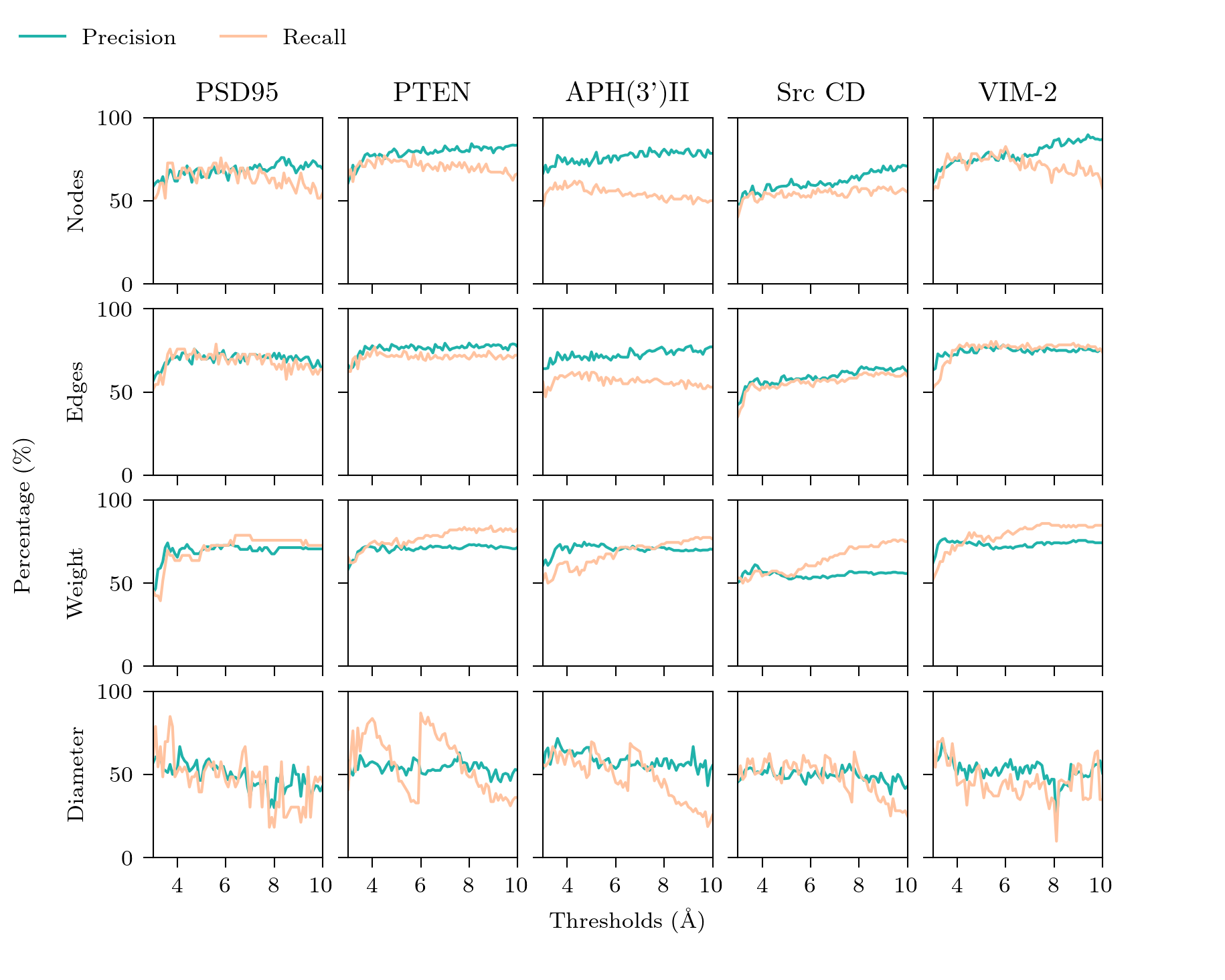

Supplement: S1 Fig — (TIF) [file pone.0261829.s001.tif]

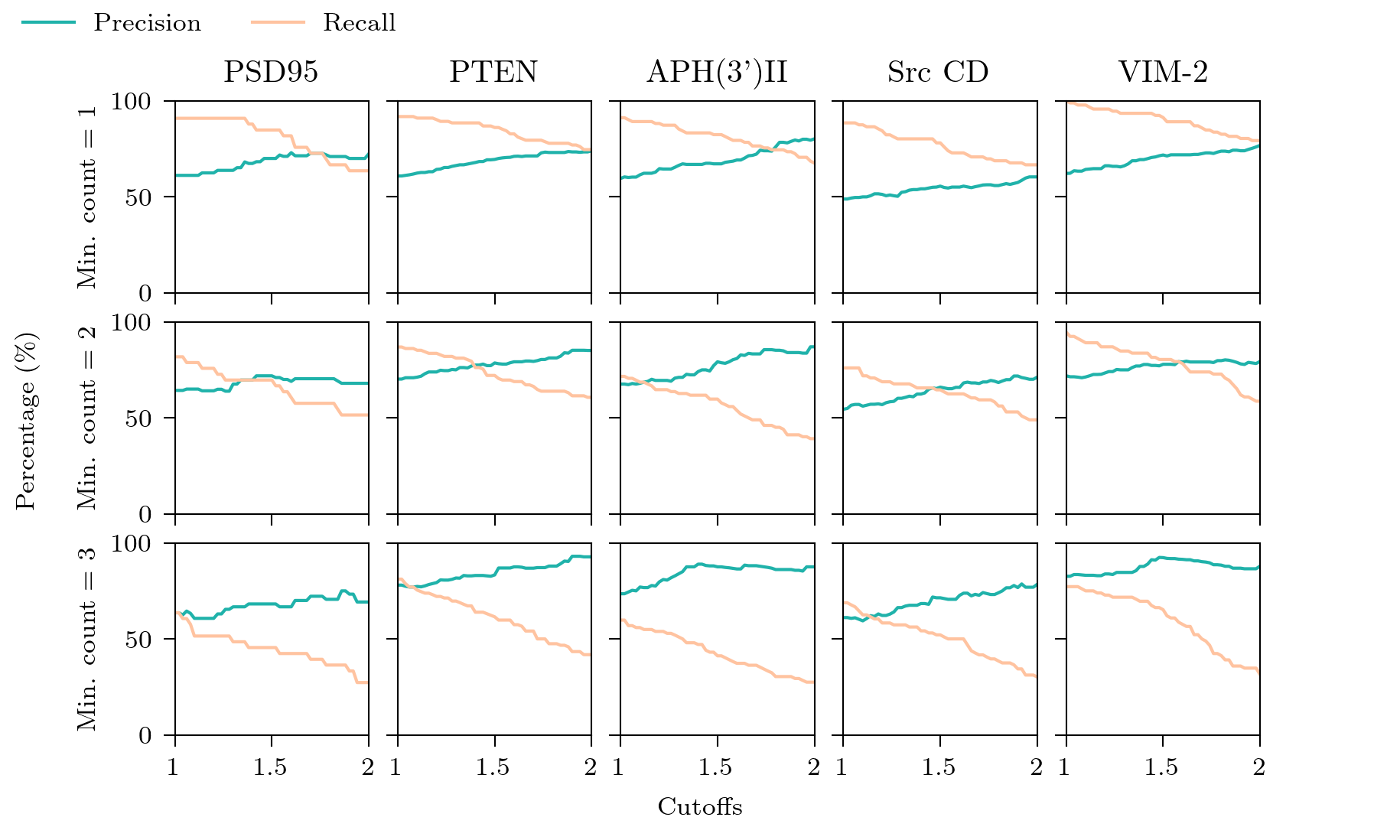

Supplement: S2 Fig — Each row and column represents a different minimum count and protein, respectively. (TIF) [file pone.0261829.s002.tif]

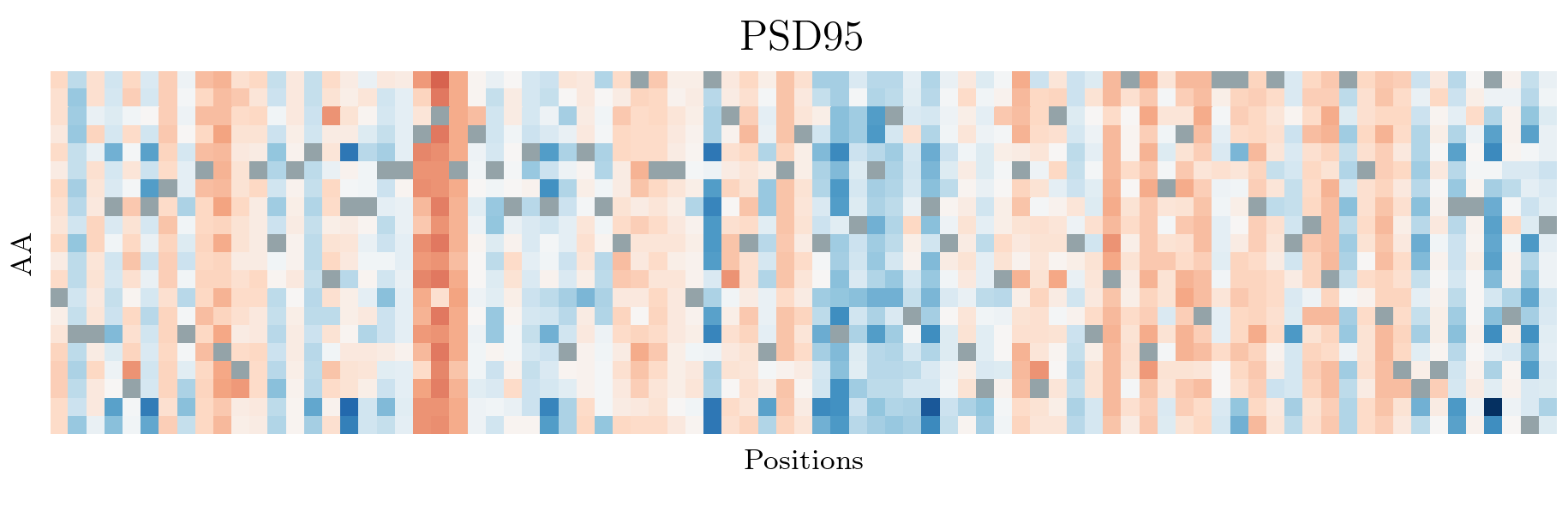

Supplement: S3 Fig — Red and blue colors represent structural loss and robustness, respectively. (TIF) [file pone.0261829.s003.tif]

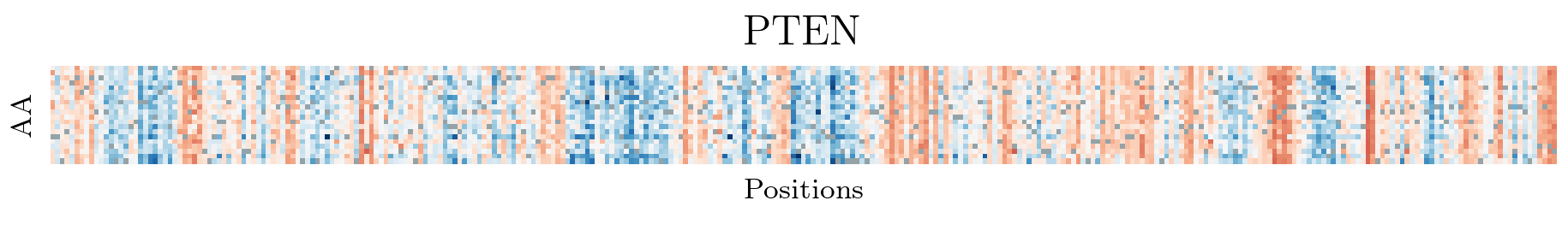

Supplement: S4 Fig — Red and blue colors represent structural loss and robustness, respectively. (TIF) [file pone.0261829.s004.tif]

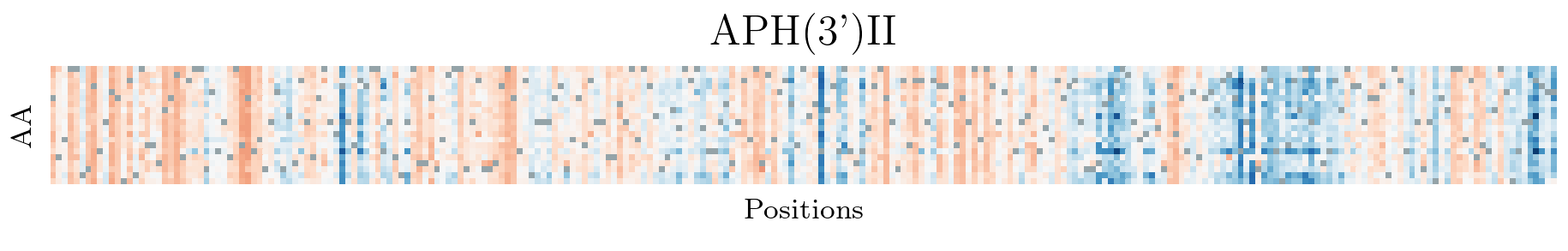

Supplement: S5 Fig — Red and blue colors represent structural loss and robustness, respectively. (TIF) [file pone.0261829.s005.tif]

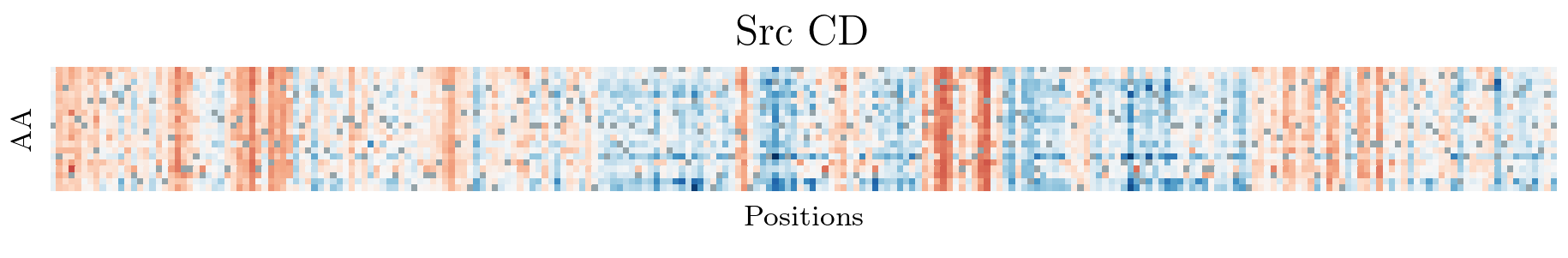

Supplement: S6 Fig — Red and blue colors represent structural loss and robustness, respectively. (TIF) [file pone.0261829.s006.tif]

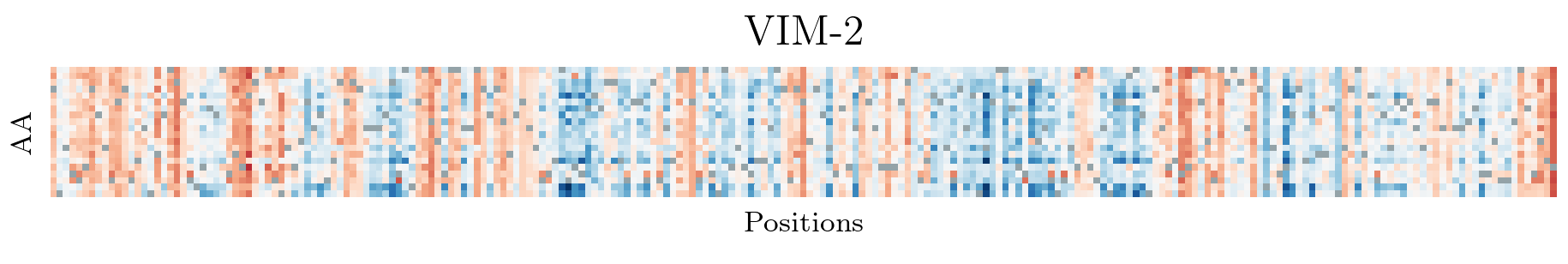

Supplement: S7 Fig — Red and blue colors represent structural loss and robustness, respectively. (TIF) [file pone.0261829.s007.tif]

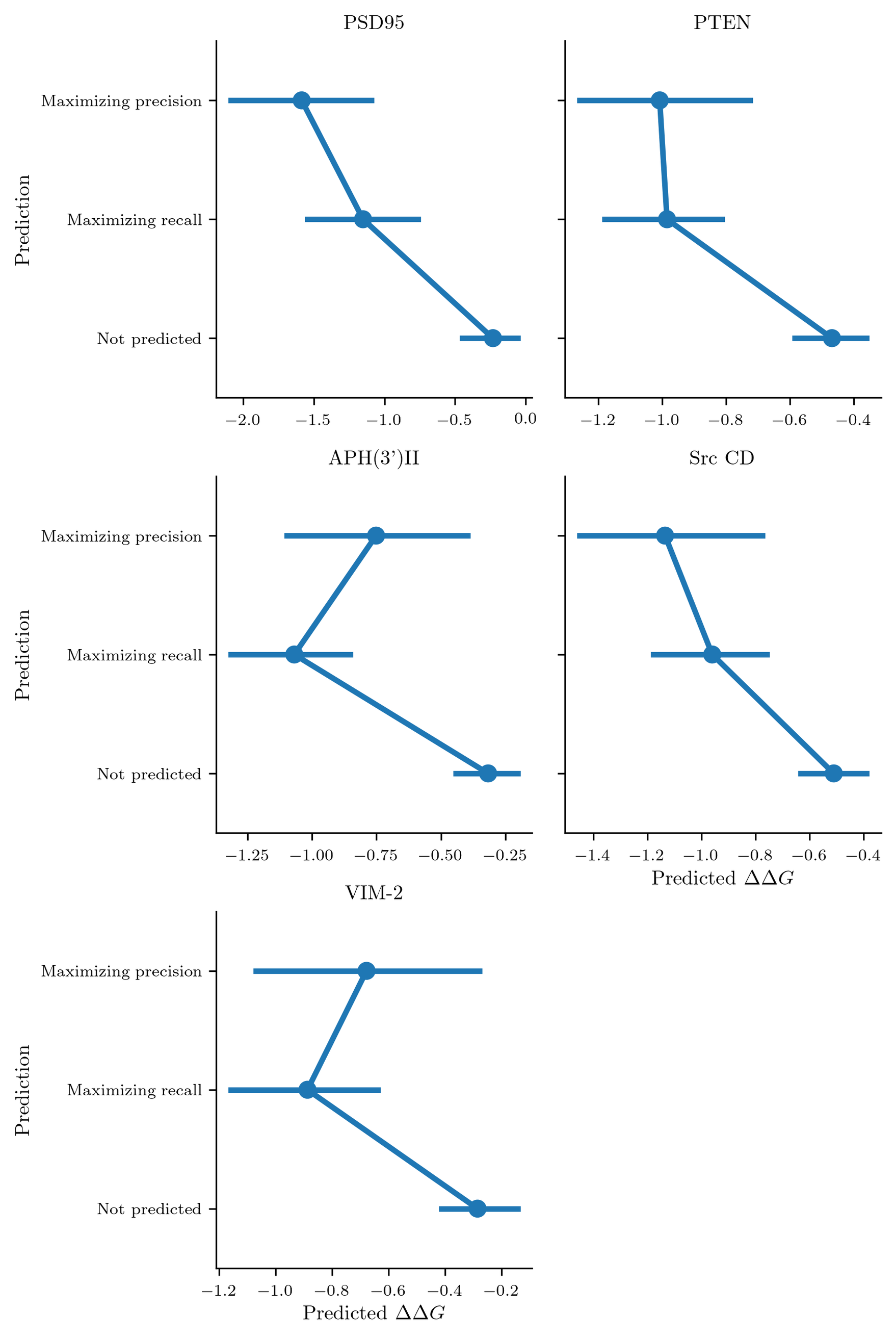

Supplement: S8 Fig — (TIF) [file pone.0261829.s008.tif]
